# A New Dental College

**Published:** 1879-06

**Authors:** 


### A NEW DENTAL COLLEGE.

The present indications are that California is soon to have a dental College that will in some respects outstrip all others in the country.

An endowment to the amount of seventy thousand dollars of productive real estate has been made by a wealthy member of the profession in San Francisco, for the establishment of a dental College in that city. Another proposes to contribute seventy thousand dollars in money for the same enterprise, making a grand total of one hundred and forty thousand dollars. This should secure a magnificent institution.

It is to be hoped that the enterprise will be carried through and an institution established that shall be thoroughly equipped in all its appointments, and be able to prosecute its work without the financial embarrassments that have to a greater or less extent attended the establishment of every similar institution in the country. California should have a good dental College; and may the enterprise be eminently successful in establishing a good standard of requirement, starting off at once on the highest level yet attained, and proceed onward and upward.

